# Casting Gold Inlays

**Published:** 1918-03

**Authors:** R. Ottolengui


### CASTING GOLD INLAYS

BY R. OTTOLENGUI, D.D.S.

Gold contracts in cooling, yet, my brother, have you not often produced inlays that would not enter a cavity, having surrounding walls because of the fact that your inlay came from the flask too large? Quite so! Yet gold contracts in cooling, does it not? What is that? The mould must have been expanded? Well, there you are!

If it be true that gold contracts when cooling, and who denies that? And if it be true that with an expanded mould, the inlay, in spite of such contraction may be larger than the wax pattern, is it not evident that there must be a method by which the mould will be expanded just sufficiently, so that the gold, contracting as it cools, assumes just the form and size of the original wax pattern? Why, of course, it must be so. Very well, then, let an ''office girl'' tell you how she does it.

''Upon receiving the wax pattern of an inlay, heat the end of a sprue former sufficiently so that it may be attached firmly to the pattern. Place it preferably at one end of the longest diameter of the pattern. Wash the surface of the wax thoroughly with acetone before attempting to place any investment. Then place the sprue former in the hole in the base and set it firmly.

''Mix the investment (Taggart's) for five minutes, constantly rotating the mixing bowl during the process. The mixing bowl should be absolutely clean, as remnants of the old investment, if left on its surface, will alter the character of the new mix, and interfere with results.

''Paint the investment onto the surface of the inlay, carefully and slowly, using a small brush. Use a thin part of the investment and coat the inlay, pushing the brush always in the same direction, thus avoiding the formation of air bubbles, which, in turn, would produce little balls, or beads, on the surface of the gold. Continue this until the investment thickens just enough to leave a complete coating on the wax. Then add more investment, taking up more on the brush and thus coating it more thickly. When the pattern is completely covered with a thick coating of the investment, put the ring in place and pour in more investment, letting it run in slowly and rise up and surround the pattern gradually till the ring is full. If any water is seen oozing from bottom of the ring, remove it gently with blotting paper.

"The invested pattern should be burned out within an hour of investing, as this stops all chemical action and avoids distortion of the mould.

"In burning out the flask it should be placed sprue hole uppermost, three inches from the top of a three-quarter-inch flame for five minutes. This should be followed by twice this amount of heat for ten minutes, or all the heat that the flask will stand without causing the wax to boil up through the sprue hole. When this occurs, steam boils with the wax and causes distortion of the mould. The last stage is to use the full flame for five minutes, or until a little flame burns out of the sprue hole.

"The exception to the above is in connection with small inlay patterns which require ten minutes at full heat. The first heating crystallizes the investment; the second draws the wax into the investment and the third burns it out. Naturally it takes a longer time for the heat to reach the space made by a small pattern than in one made by a large pattern.

"Time the burning out of the invested inlays with an alarm clock. Such clocks may be had, which can be set for five, ten or more minutes. The ringing of the alarm will remind you to change the flow of gas for the next stage. Full directions for setting the clock come with it.

"To remove the base and sprue former heat slightly over the flame. Hold the flask with sprue hole downwards.

This will avoid all possibility of dropping a scrap of investment into the pattern cavity.

"Never use the remnant of a previous casting for making a new inlay without thoroughly refining the gold. It really pays never to use gold a second time, as gold can not be refined as well by a dentist as by an expert gold refiner. Gold can be obtained in nugget form, and a fresh nugget used for each casting. The remnants can be returned to the gold dealer who will give credit for them. The small difference between the buying and the selling price of these gold nuggets is more than covered by the advantage of knowing that your gold is always right for casting. Anyway, never use 'office scraps,' trying to refine them yourself. If you do, you are not using identical methods and materials all the time, and of course will not get even results. Therefore always use the same grade or karat of gold.

"In casting, avoid overheating the flask. Arrange the flame so as to have a perfect mixture of gas and nitrous oxide. There should be no smoking. The flame should be short, blue and very intense. This will boil the gold without unduly heating up the flask. Having found the right flow of illuminating gas, it would be a good idea to set a stop-pin in the gas outlet so that it could always be opened to just the same flow. Then open the valve until six pounds of nitrous oxid is registered. As soon as the gold actually boils, make the cast with a rapid throw of the lever.

"To thoroughly cleanse the gold leave it in hydrofluoric acid over night."—*Dental Items of Interest* for November.

